# Case Report: Robotic Repair of a Perineal Hernia Following Abdominoperineal Resection

**DOI:** 10.3389/jaws.2024.13481

**Published:** 2024-11-29

**Authors:** Madison Brune, Austin Hotop, William Silliman, Kevin Bartow

**Affiliations:** ^1^ Department of General Surgery, University of Missouri, Columbia, MO, United States; ^2^ Department of General Surgery, St. Francis Medical Center, Cape Girardeau, MO, United States

**Keywords:** perineal hernia, robotic surgery, rectal cancer, abdominoperineal excision, extralevator abdominoperineal excision

## Abstract

**Introduction:**

Perineal hernias, protrusions through the pelvic diaphragm, are a rare complication post-abdominoperineal resection. The shift to extralevator APR techniques could be linked to a potential increase in these hernias. This case series evaluates the surgical management of perineal hernias, focusing on the evolving role of robotic surgery. Given the limited existing research on robotic repairs in this context, it highlights its potential as an innovative approach.

**Presentation of Case:**

In a case series, we report three patients who underwent robotic abdominoperineal resection (APR) for rectal and anal canal carcinoma after neoadjuvant chemoradiation. The 65-year-old female developed a perineal hernia 7 months post-operatively, the 67-year-old male after 4 years, and the 63-year-old female presented with a recurrent perineal hernia post-APR with gracilis flap reconstruction. All patients underwent successful robotic hernia repairs with mesh placement and demonstrated symptomatic improvement post-operatively.

**Discussion:**

Perineal hernia management lacks a standardized protocol, with methods ranging from open to laparoscopic techniques. A review of recent literature suggests increasing favorability towards laparoscopic and robotic approaches due to their less invasive nature. Our cases demonstrate the advantages of robotic surgery’s precision and improved visualization, supporting its use in perineal hernia repair, although more research is needed to confirm.

**Conclusion:**

Robotic-assisted surgery for perineal hernia repair post-APR shows promise, enhancing the benefits of laparoscopic methods. This series underlines the potential of this approach, though further investigation in larger studies is essential to establish its advantages.

## Introduction

Perineal hernias can be defined as the protrusion of intraperitoneal or extraperitoneal contents through a congenital or acquired defect of the pelvic diaphragm into the perineum [1]. The occurrence of post-operative perineal hernia after abdominoperineal resection (APR) is rare, with incidence reported below 1% [2]. However, a growing body of evidence suggests its true incidence may be higher because of under-reporting and technical updates. In general, perineal hernias proceed asymptomatically, which, unfortunately, results in a large number of unreported cases [3]. Additionally, there is concern that the rate may be increasing due to the growing popularity of extra levator APR, which, despite its oncological advantages, may lead to more significant pelvic defects and, subsequently, higher rates of hernia occurrence [4, 5]. Two larger studies by West et al. and Sayers et al. reviewed the frequency of perineal hernias post-extra levator abdominoperineal excision (eAPR). They reported frequencies of 2.8% and 26%, respectively, highlighting the variability of reported incidences [6, 7].

The scarcity of perineal hernias has limited the scope of large-scale studies, leaving the majority of treatment guidelines to be derived from case reports and small series. Asymptomatic perineal hernias are often managed conservatively; however, when symptomatic, presenting with discomfort, bulging, or complications such as urinary dysfunction, intestinal obstruction, or skin erosion, surgical intervention becomes necessary [5].

Recent literature has increasingly examined the merits of laparoscopic versus open-surgical approaches for perineal hernia repair. This case series contributes to the discussion by summarizing the advantages of laparoscopic techniques and exploring their integration with robotic surgery. Three cases of perineal hernia repairs performed post-APR between 2020 and 2024 were included in this series. The report delves into the shared benefits and distinct strengths of robotic surgery in pelvic procedures and hernia repairs, applying these insights to perineal hernia management. Notably, the literature reveals only two prior cases of robotic repair post-APR, underscoring the novelty and potential of this approach [8, 9].

## Case Presentation

### Case 1

A 65-year-old woman with a history of malnutrition and smoking presented to the clinic to discuss the surgical resection of her rectal cancer. She had completed neoadjuvant chemoradiation prior to her visit. The case was deliberated with the referring oncologist, and after thorough discussions with the patient and her family, it was decided to proceed with robotic abdominoperineal resection (APR). The surgery was executed without intraoperative complications, and subsequent pathology revealed a T2 tumor invading the muscularis propria with clear resection margins.

Seven months post-APR, the patient reported a tender, fluid-filled swelling at the perineal closure site. Physical examination revealed a cystic fluid collection and surgical drainage was recommended. A non-diagnostic CT scan was conducted, and the patient consented to the surgical drainage procedure. Amber fluid was evacuated, the area was examined for further abnormalities, and the site was closed.

**FIGURE 1:**
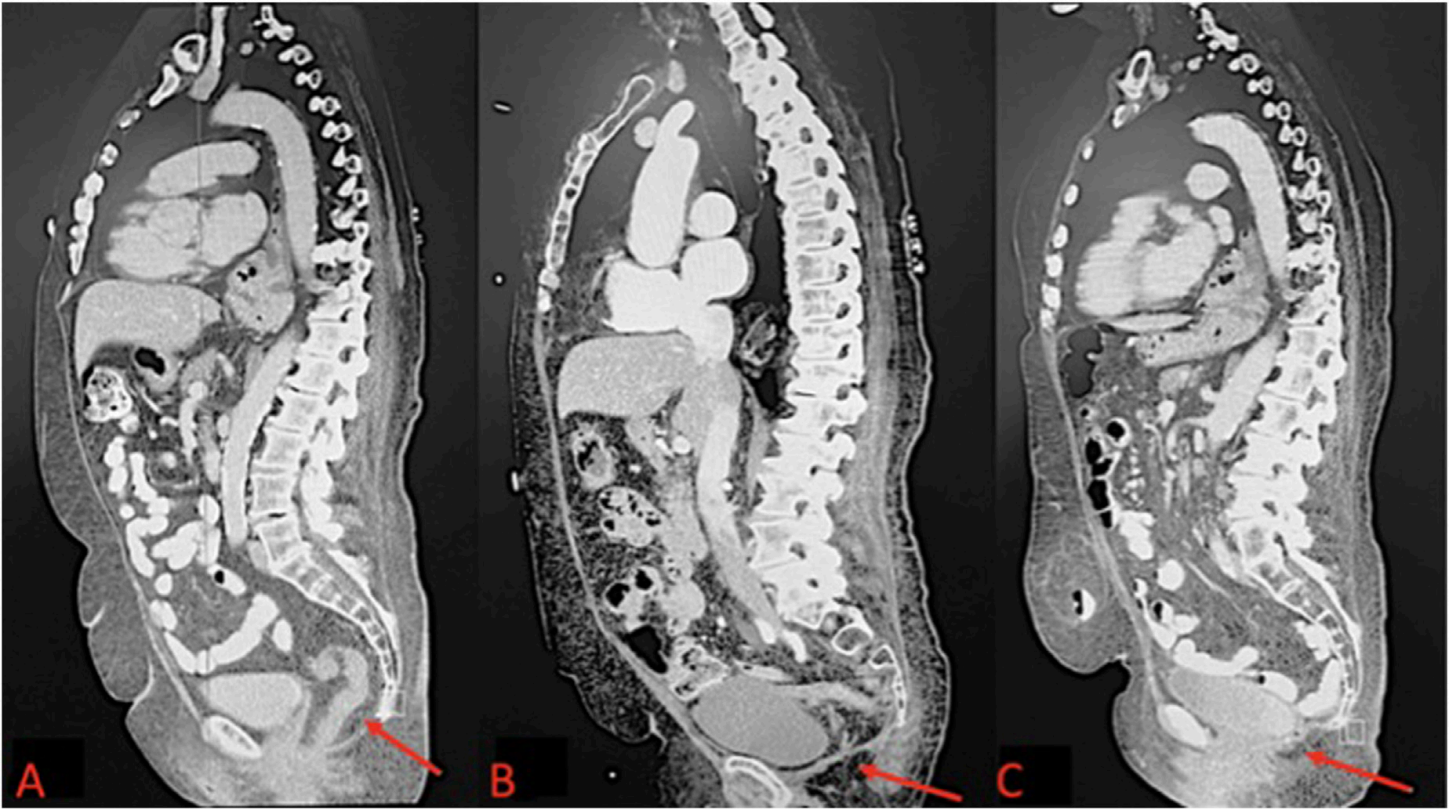
**(A)** Pre-operative CT scan of perineal hernia (red arrow); **(B)** One month post-operative CT image showing surgical correction of perineal hernia; **(C)** One year post-operative CT Image.

Approximately 1 year after the APR, the patient complained of a painful bulging in her perineum that improved when lying down but worsened upon standing or moving. She specifically noted that she could feel her intestines descend into the hernia sac, and she was manually able to reduce the hernia with pressure. A CT scan with oral and IV contrast was performed to distinguish between a perineal hernia and seroma. The imaging confirmed a perineal hernia with incarcerated small bowel and omentum. She was counseled about her diagnosis and treatment options, and she opted for robotic perineal hernia repair with mesh insertion (Figure 1A).

The patient underwent general anesthesia and was placed in the lithotomy position. Abdominal access was achieved via the Hasson technique in the right upper quadrant, and pneumoperitoneum was established. The camera was then introduced into the abdomen. 8mm trocars were placed in the upper quadrants bilaterally, followed by placement of a 12 mm trocar into the right upper quadrant, and the perineal hernia was identified containing small bowel and omentum. The contents were reduced, and adhesiolysis was performed to free adhesions. The hernia defect was sutured closed with a running 2-0 V-Lock stitch. A 4.5-inch Sepramesh IP Composite polypropylene mesh was selected and secured over the defect using a running 2-0 V-Lock suture. Absorbable tacks were used for additional mesh anchoring. Post-reduction, the abdomen was inspected, ports were removed, and the pneumoperitoneum was released. The incisions were closed with a 4-0 Monocryl in a subcuticular pattern. Postoperatively, the Early Recovery After Surgery (ERAS) protocol was followed to manage the patient’s pain, advance her diet, and encourage early ambulation prior to discharge. One month after surgery, the patient reported gradual pain relief and complete resolution of the perineal sliding and bulging symptoms. Imaging performed at the 1-month follow-up confirmed that the hernia repair was successful (Figure 1B). The patient was then evaluated at 2 months, 6 months, and 1 year postoperatively, with imaging performed at the 1-year visit to assess for recurrence (Figure 1C).

### Case 2

A 67-year-old male initially presented to the gastroenterology clinic with rectal bleeding and concerns of hemorrhoids. A subsequent examination revealed a 6 cm rectal tumor, prompting a referral for surgical evaluation. At that time, his carcinoembryonic antigen (CEA) level was less than 1, and a CT scan indicated no metastasis. An MRI of the pelvis showed a low-lying rectal tumor with a cranio-caudal extent of approximately 4.4 cm. Following neoadjuvant chemoradiation, the patient underwent a robotic abdominoperineal resection (APR) due to the tumor’s proximity to the anal area.

The operation proceeded without intraoperative complications, and pathology confirmed a T3N2M0 tumor extending through the muscularis propria into the perirectal fat. There was no involvement of the anal sphincters or mesorectal fascia, and clear margins were achieved. Later in the same month, the patient developed a perianal abscess, necessitating wound debridement. Two years post-operatively, a parastomal hernia presented as a large bulge at the ostomy site. The hernia was successfully repaired using robotic-assisted mesh placement.

**FIGURE 2:**
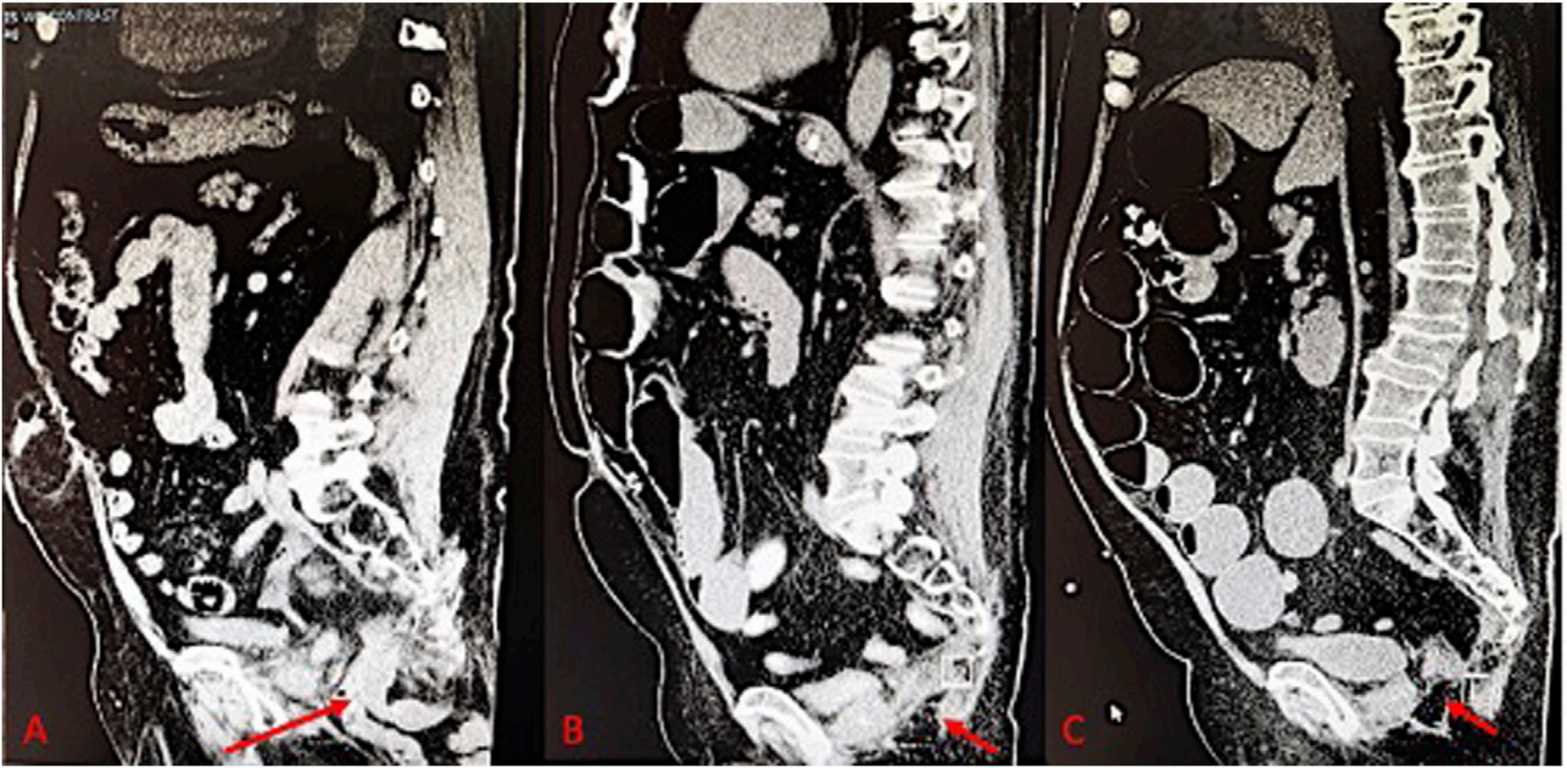
**(A)** Pre-operative CT scan of perineal hernia (red arrow); **(B)** One month post-operative CT image showing surgical correction of perineal hernia; **(C)** One year post-operative CT Image.

Four years after the APR, the patient experienced perineal swelling diagnosed as a perineal hernia, accompanied by a sensation of visceral prolapse and the ability to reduce the herniation manually (Figure 2A). A laparoscopic robotic-assisted repair was performed. Under general anesthesia, in the prone position, an incision was made over the prior perineal scar, and dissection revealed a hernia sac from which multiple loops of small bowel were reduced. The 5 cm × 5 cm defect was repaired with a 12 cm round Ventralex mesh, secured with PDS sutures, and the site was drained from the upper gluteal region. The closure included interrupted Vicryl sutures, a running 4-0 Monocryl, and a Dermabond dressing.

The patient was repositioned from prone to supine, allowing for mid-abdominal access through the rectus muscle, where a balloon trocar was placed for CO_2_ insufflation. Camera inspection identified adhesions to the prior mesh. After adhesiolysis and bowel reduction, two additional robotic trocars facilitated the docking of the robot. The mesh was secured to the peritoneum with 2-0 V-Loc sutures, and the fascia and skin were closed with 0 Vicryl and 4-0 Monocryl, respectively. Postoperatively, the ERAS protocol was followed to manage the patient’s pain, advance his diet, and encourage early ambulation prior to discharge. One month after surgery, the patient reported gradual pain relief and resolution of the hernia-related symptoms. A CT of the abdomen and pelvis was performed at the 1-month follow-up to confirm the successful repair of the perineal hernia (Figure 2B). The patient was then evaluated at 2 months, 6 months, and 1 year postoperatively, with imaging performed at the 1-year visit to assess for recurrence (Figure 2C).

### Case 3

A 63-year-old female with a significant medical history of anal canal carcinoma initially treated with chemoradiation and robotic-assisted abdominoperineal resection with gracilis flap reconstruction presented for surgical evaluation. She has a history of hyperthyroidism, malignant neoplasm of the anal canal, and obesity. The patient also had advanced squamous cell carcinoma (SCC) of the anus following the Nigro protocol with radiation therapy and chemotherapy. Complications included a local recurrence leading to a malignant rectovaginal fistula, requiring resection and post-operative challenges such as recurrent urinary tract infections, radiation cystitis, and urethral stricture. Subsequent surgical interventions included a repair of the pelvic floor hernia and presentations of incisional and parastomal hernias.

The patient underwent a perineal hernia repair via a transperineal approach. Entry into the presacral space revealed bowel and omentum, which were reduced and resected, respectively. A pelvic defect was identified, partially obstructed by the uterus anteriorly. It was closed by securing a mesh from the coccyx to the top of the uterus, reinforced by an additional layer before multiple-layer pelvic soft tissue closure.

**FIGURE 3:**
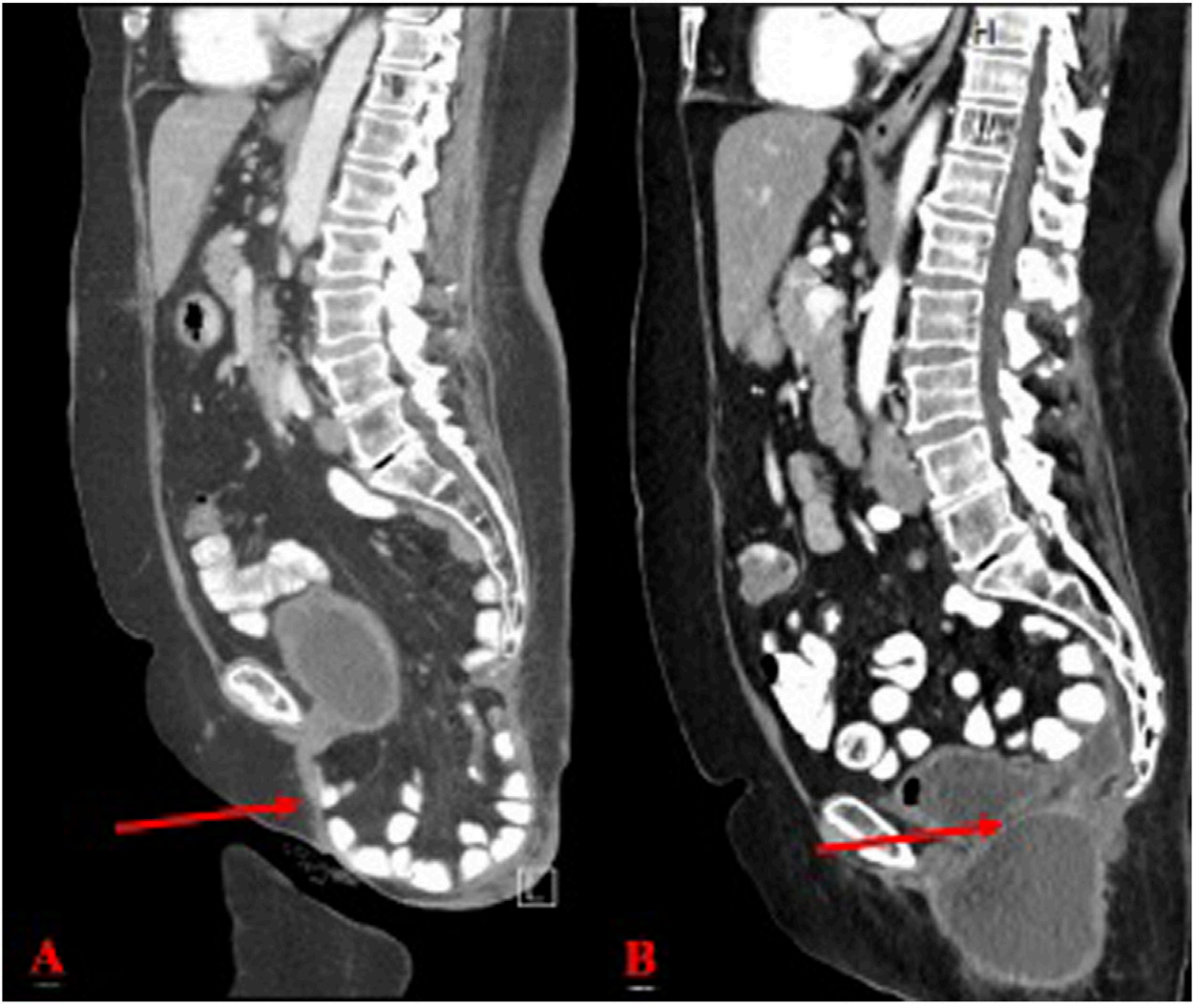
**(A)** Pre-operative CT scan of perineal hernia (red arrow); **(B)** One month post-operative CT image showing surgical correction of perineal hernia.

She underwent a diverting sigmoid ostomy followed by APR surgery for a recurrence. She completed additional chemoradiation and was on Eliquis for a left femoral vein DVT. Recurrent UTIs and persistent perineal hernia pain, rated at 5/10 in severity, were also noted. The hernia’s size remained stable and was reducible with position changes (Figure 3A).

She had an open perineal hernia repair with mesh placement, partial omentectomy, and adhesiolysis. Despite these efforts, imaging later revealed a parastomal hernia with associated complications. The hernia had recurred, prompting further assessment and surgical planning. The most recent surgical intervention involved extensive adhesiolysis and hernia repair with a 15 cm × 12 cm Ventralight mesh placement via a robotic anterior approach. A distal appendiceal mass, suggestive of a mucinous neoplasm, warranted an appendectomy. The operative course included repositioning to prone for excision of the hernia sac and drainage of the perineal space, with meticulous attention to hemostasis and closure. She developed paralytic ileus postoperatively with nausea and vomiting, requiring NG decompression. Her diet was gradually advanced, and she tolerated regular food. Following the ERAS protocols, her Foley catheter and NG tube were removed, and her pain was well controlled. After successful ambulation and clearance by PT, she was deemed stable for discharge 7 days post operatively.

At her 1-month post-operative follow-up, she reported no significant complaints other than pain while sitting, which she felt was manageable. Examination of the perineal incision revealed appropriate healing and the presence of subcutaneous fluid collection, consistent with an expected seroma (Figure 3B). Stitches were removed without complications. Patient was seen at 2 months and 6 months post operatively with 1 year follow up scheduled.

### Patient Perspective

The three patients in this case series provided their perspectives on the treatments they received, highlighting various outcomes and levels of satisfaction. Patient 1 reported doing well, expressing happiness with the surgery and no longer experiencing the bulging sensation while sitting. Patient 2 mentioned overall improvement but continued to experience perineal swelling due to the hernia, though the ostomy was functioning appropriately, and they denied any pain. Patient 3 had no significant complaints, aside from some pain while sitting, and felt that their bottom was intact. They also denied experiencing fevers, chills, or night sweats and overall felt well, with a functioning ostomy. Each patient signed consent forms prior to their procedures, agreeing to have their cases documented or presented.

## Discussion

Perineal hernia represents a complex post-operative complication following abdominoperineal resection (APR) and continues to pose a challenge for surgeons. While the incidence of perineal hernia post-APR is traditionally considered to be low, specifically under 1%, recent evidence suggests that this figure is an underestimate [2]. Surgical management of rectal carcinoma has evolved in recent years towards minimally invasive techniques, such as the extralevator abdominoperineal excision, which involves the removal of the entire pelvic floor muscle complex. Additionally, neoadjuvant and adjuvant chemotherapy are becoming more common [10]. These modifications to treatments collectively provide an improved oncological outcome; however, these changes could also contribute to an increased incidence of perineal hernias [6].

Surgical modifications have led to a greater likelihood of the small intestine descending into the pelvic region. This has been reflected in the reported incidence of post-APR perineal hernias, which some studies suggest is between 2.8% and 26%. However, the actual rate may be even higher due to the underreporting of minor and asymptomatic hernias [6, 7]. Factors implicated in this increased incidence include the creation of larger pelvic floor defects, necessitating reliance on the weaker ischioanal fat and skin for defect closure, and a diminished rate of post-operative adhesion formation. Additional risk factors identified in the literature for perineal hernia include post-operative wound infection, pelvic radiotherapy, female gender, and obesity [3].

The surgical management of perineal hernias lacks a consensus regarding the optimal approach. Current approaches for hernia closure include open surgery with perineal, abdominal, or combined techniques and laparoscopic surgery with an abdominal approach. A systematic review of 30 studies, spanning a decade from 2012 to 2022, reveals various practices in addressing perineal hernia repairs. The laparoscopic method was employed in 36.7% of cases, open abdominal in 16.7%, a combined abdominoperineal technique in 26.7%, a strictly perineal approach in 13.3%, and a robot-assisted abdominal approach in 6.7% of the reported instances [11].

Open perineal and abdominal methods offer substantial exposure for mesh placement and suturing but carry an elevated risk of wound complications and infections due to their invasiveness [9]. In contrast, the laparoscopic abdominal approach is gaining favor for its superior intra-abdominal visualization, potential for assessing tumor recurrence, and obviation of an additional perineal incision, as opposed to perineal or open abdominal techniques [12]. However, it also presents challenges such as limited pelvic space and difficulties in mesh securing [13].

In this case series, the use of an abdominal robotic approach retained the advantages of the laparoscopic method while adding the unique benefits of robotic surgery. Robotic surgery, especially pertinent to pelvic operations, offers enhanced three-dimensional visualization, improved maneuverability, stability, ease of suturing, precise mesh positioning, and access to otherwise difficult areas [14–16]. Each feature is particularly advantageous when navigating the restricted space and complex anatomy of the pelvis.

In this case series, different approaches were taken in terms of patient positioning based on the complexity and size of the hernia. One of the patients was placed in the prone position, as this case involved a larger hernia that raised concerns about the ability to adequately close the fascia using a strictly robotic approach. Prone positioning allowed for better access and improved fascial closure in this more complex case, although it did result in a longer operative duration. In contrast, the other patients were managed in the supine position, which was deemed sufficient given the smaller size of the hernias and less challenging anatomy. This variation in patient positioning highlights the importance of tailoring the surgical approach to the specific characteristics of each case, ensuring optimal outcomes for each patient.

Although there are no readily available studies comparing the outcomes of laparoscopic vs. robotic repair of perineal hernias, numerous studies have highlighted the advantages of robotic surgery in various hernia repairs, such as shorter hospital stays, decreased rates of surgical site complications, and improved fascial closure rates [3, 17]. These advantages are theoretically applicable to perineal hernia repairs as well. However, a notable limitation of robotic surgery is the increased time required for the procedure [3, 5, 14, 17]. In the present cases, the potential benefits of robotic repair were deemed to justify the longer operative duration.

Choosing an appropriate repair technique is critical to minimize the risk of hernia recurrence. Recent systematic reviews indicate that perineal hernia repairs have a 22% recurrence rate, underscoring the need for carefully selected surgical strategies [18].

## Conclusion

This report has detailed three successful robotic-assisted repairs of a perineal hernia following an abdominoperineal resection (APR). Follow-up assessments indicated a favorable outcome, with the absence of hernia recurrence in all three cases. The integration of robotic assistance in the repair of perineal hernias has the potential to enhance the already established benefits of laparoscopic techniques, taking advantage of the proficiency robotic surgery has shown in the broader domain of hernia and pelvic surgeries. While research remains limited due to small sample sizes, larger cohort studies are warranted to validate efficacy and benefits, ultimately moving toward standardized protocols for robotic-assisted perineal hernia repair.

## Data Availability

The original contributions presented in the study are included in the article/supplementary material, further inquiries can be directed to the corresponding author.
